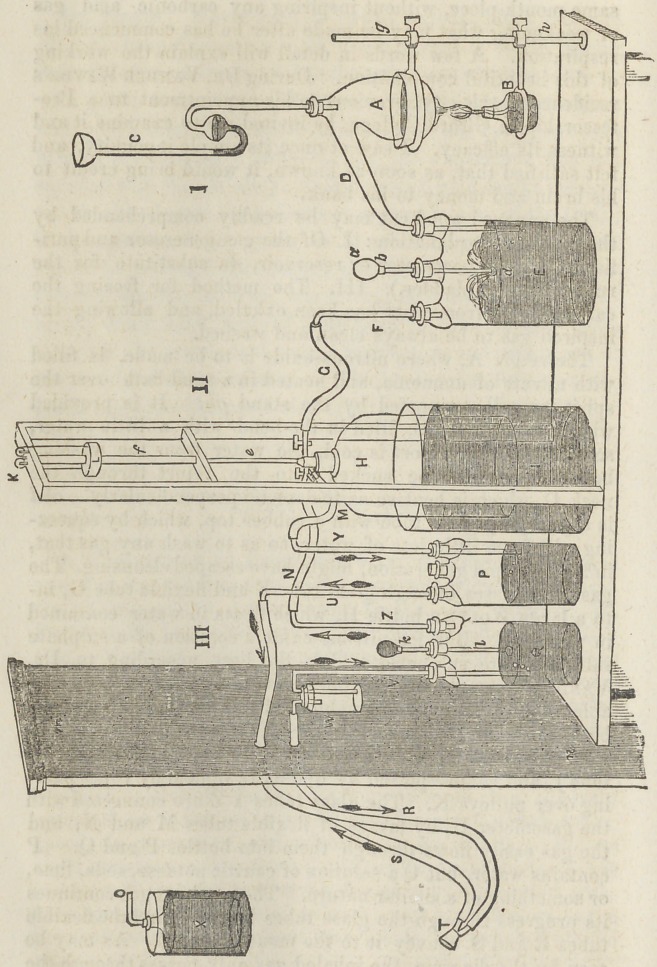# A Description of Professor Vander Weyde’s Apparatus, for the Generation and Administration of Nitrous-Oxide and Other Anæsthetic Gases

**Published:** 1866-08

**Authors:** Samuel W. Francis

**Affiliations:** Fellow of the New York Academy of Medicine


### Selections.

A Description of Professor Vander Weyde's Apparatus, for the Generation and Administration of Nitrous-Oxide and other Anæsthetic Gases.—By Samuel W. Francis, M. D., Fellow of the New York Academy of Medicine.—Many practioners would administer laughing gas, were it not for the cumbrous and offensive india-rubber bag now in use. It is much easier to place a patient under the influence of an anæsthetic by means of a few drops of chloroform, or even with the temporary delay of ether, than to subject him to the annoyances incident to the present use of breathing-bags. But Professor Vander Weyde has recently patented an invention easy of use, durable in construction, speedy in generation, and effective in its results. By its rapid arrangements, nitrous-oxide can be made the instant it is required; retained in reservoir for an indefinite period, without degeneration or loss of volume; and what is more to the point of interest, the patient may inhale and exhale from the

*The last page of the manuscript of this report, containing a few paragraphs, was lost. It contained no material point, however.—[Ed.

same mouth-piece, without inspiring any carbonic acid gas or weakening what may be made after he has commenced his respiration. A few words in detail will explain the working of this beautiful combination. During Dr. Vander Weyde's residence in this city, previous to his appointment to a Professorship in Girard College, he invited me to examine it and witness its efficacy. I saw at once its simple ingenuity, and felt satisfied that, as soon as known, it would bring credit to his brain and money to his bank.

The annexed wood-cut may be readily comprehended by the following explanation: I. Of the gas generator and purifier. II. The gasometer or reservoir, (a substitute for the rubber bag or bladder.) III. The method for freeing the carbonic acid from what has been exhaled, and allowing the inspired gas to be always clean and washed.

The retort A, where nitrous-oxide is to be made, is filled with nitrate of ammonia, and heated in a sand-bath over the spirit-lamp B, supported by the stand *gh*. It is provided with a safety tube, C, filled in the bend with a little water, so that when the retort is cool, the water from the washing bottle E, cannot be sucked into the retort through the neck D, which is bent so as to descend perpendicularly. *abd* is a purifying glass tube with a rubber top, which by squeezing will force little jets of water, so as to wash any gas that, in its too rapid generation, might have escaped cleansing. The gas then passes through glass tube F and flexible tube G, into a large Wolfe's bottle H, which floats in water contained in vessel I. But for nitrous-oxide a solution of a sulphate salt or dilute sulphuric acid is the best, according to Dr. Vander Weyde's many experiments; for pure water is capable of absorbing its own volume of this gas, and if the vessel be not very strong, the atmospheric pressure would crush it. The gasometer H, is balanced by guiding-rod *e*, sliding in tube *f*, and counterpoised by weight L, upheld by ropes passing over pulleys K. The glass tubes Y Z are connected with the gasometer H, by means of flexible tubes M and N, and the gas easily flows through them into bottles P and Q. P contains water, but Q a solution of caustic potassa, soda, lime, or something of a similar nature. The laughing gas continues its progress through the glass tubes U and V, and the flexible tubes R and S convey it to the mouth-piece T. As may be seen by the diagram, the inhaled gas only passes through the water in bottle P, while that exhaled is forced through the

I
II
III
A
B
C
D
E
F
G
H
K
L
M
N
O
P
Q
R
S
T
U
V
W
X
Y
Z
a
b
d
e
f
g
h
k
l
m
n solution of potassa, which deprives it of the carbonic acid gas. Bottle Q may have a glass tube and rubber in *abd* in bottle E, to send out jets and more effectually wash the gas if desirable. The beautiful part of the mechanical portion of this invention is the purifying arrangement. The gas is allowed to pass but in one way, for tubes V and Y plunge under the liquids in bottles Q and P. Hence the gas only passes downward in them. In the upward action, the liquids rise 2, 4, or 6 inches, and thereby act as complete valves. Any other kind of valves in the lower end of tubes V and Y, would not produce as favorable a result. The direction of flow may be seen by the arrows.

As an additional safety in preventing any liquid by any possibility being sucked into the mouth, owing to the great excitement, at times, of the patient, Prof. VANDER WEYDE places a small vessel W, between V and S, so that on a strong inspiration, it would pour into W, but on the next expiration it would be blown back into the bottle Q, and thereby all danger in that quarter is prevented. It is impossible, in detailing a description where letters are employed, and one has not seen the apparatus in motion, for it works very like a little steam engine, to suit unmechanical minds. For there is apt to exist an appearance of confusion or complication that does not in any way form a part of the true instrument. But if this plain method were once seen, it would convince the most skeptical, and especially those who have been accustomed to use the big bag or bladder, which soon becomes foul by exhaled carbonic acid gas.

The small jar X, on the left, may also be used as an artificial respirator, by attaching tube O to mouth-piece T, and moving it up and down.

For fear that unruly and over-excited patients might destroy this glass apparatus, Prof. VANDER WEYDE suggests passing the flexible tubes S R through a wall or partition *mn*, so that it may be in one room, and the gentleman breathing laughing gas in another—this dispenses with the last objection that can be made.

For hospitals it would prove most excellent, as the entire apparatus could be kept in a separate room ; the gas made when necessary, and, if not used, held in reservoir for an indefinite period. As nitrous-oxide has been found by actual experience to be by far the least injurious of all anæsthetics, it would seem advisable for this subject to be thoroughly investigated, now that a suitable contrivance has been made.

The following remarks of Prof. VANDER WEYDE, are worthy of consideration, as coming from a philosophical chemist:

"It is a singular fact, that among the anæsthetics now in use—ether, chloroform, and nitrous-oxide — the first is combustible in itself, though the very *opposite* of a supporter of combustion and life; the second, chloroform, is neither combustible nor a supporter of combustion; while the last, nitrous-oxide, *is a powerful supporter of combustion and life.*" There is much worthy of the profoundest reflection in this sentence, if it be the aim of the practitioner not only to remove shattered limbs and alleviate suffering, but also to render as safe as possible the condition of his patient while undergoing an operation.

Prof. VANDER WEYDE intends to erect a factory for the purpose of preparing nitrous-oxide in liquid form, and supplying hospitals with small cylinders of it, condensed under a pressure of nearly 50 atmospheres. This will enable physicians to keep large quantities on hand in a small compass, as is the case with soda-water, especially as it is not explosive, for the liquid does not suddenly take the gaseous form.—*Med. & Sur. Rep.*

## Figures and Tables

**Figure f1:**